# Progranulin deficiency in Iba-1^+^ myeloid cells exacerbates choroidal neovascularization by perturbation of lysosomal function and abnormal inflammation

**DOI:** 10.1186/s12974-021-02203-1

**Published:** 2021-07-25

**Authors:** Kei Takahashi, Shinsuke Nakamura, Wataru Otsu, Masamitsu Shimazawa, Hideaki Hara

**Affiliations:** 1 Molecular Pharmacology, Department of Biofunctional Evaluation, Gifu Pharmaceutical University, 1-25-4 Daigaku-nishi, Gifu, 501-1196 Japan; 2 Department of Biomedical Research Laboratory, Gifu Pharmaceutical University, 1-25-4 Daigaku-nishi, Gifu, 501-1196 Japan

**Keywords:** Progranulin, Microglia, Macrophage, Lysosome, Inflammation, Neovascularization

## Abstract

**Background:**

Age-related macular degeneration (AMD) is the principal cause of permanent blindness among elderly individuals worldwide. Chronic inflammation in the subretinal space is associated with a progression of exudative AMD. Progranulin (PGRN) is a growth factor secreted from myeloid cells and plays an important role in controlling the lysosomal function. A deficiency in PGRN leads to inflammation of the neurons in the central nervous system. The purpose of this study was to investigate the role played by PGRN in the size of the choroidal neovascularization (CNV) in laser-induced CNV mice.

**Methods:**

CNVs were induced in C57BL/6J mice by laser photocoagulation of the retina. The expression of PGRN and the accumulation of Iba-1^+^ cells around the sites of the CNVs were determined. *Grn*^*−/−*^, *Grn*^*+/−*^, and *Grn*^*+/+*^ mice with laser-induced CNVs were also studied. To evaluate the effect of macrophages on the inflammation, we used a macrophage cell line (RAW264.7) in which the expression of PGRN was knocked down by RNA interference and peritoneal macrophages derived from *Grn*^*−/−*^ and *Grn*^*+/+*^ mice. These cells were incubated under hypoxic conditions (1% O_2_).

**Results:**

Iba-1^+^ myeloid cells migrated and accumulated in the photocoagulation-induced CNV areas, and the CNV lesions secreted high levels of PGRN in *Grn*^*+/+*^ mice. The size of the CNVs was larger in *Grn*^*−/−*^ mice than in *Grn*^*+/−*^ and *Grn*^*+/+*^ mice. In *Grn*^*−/−*^ mice, the number of ocular-infiltrating Iba-1^+^ cells around the CNV was higher, and these cells produced more VEGF-A than the cells in the *Grn*^*+/+*^ mice. PGRN-silencing of RAW264.7 cells led to abnormal activation of the cells. In addition, hypoxic conditions promoted the production of proangiogenic and proinflammatory cytokines from PGRN-deficient macrophages. Interestingly, the expression level of lysosome-associated proteins and the number of activated lysosomes increased in PGRN-deficient macrophages.

**Conclusions:**

These findings indicate that PGRN deficiency in Iba-1^+^ cells activates the lysosomal function that then leads to abnormal inflammation. The aberrant activation of Iba-1^+^ myeloid cells might contribute to the progression of the CNV and the regulation of these cells might be a novel therapeutic target for exudative AMD.

**Supplementary Information:**

The online version contains supplementary material available at 10.1186/s12974-021-02203-1.

## Introduction

Age-related macular degeneration (AMD) is the principal cause of permanent blindness and visual disability among individuals over 60 years of age throughout the world [1]. Early AMD is usually asymptomatic even though a mottling of the retinal pigment epithelium (RPE) and extracellular drusen deposits are present between the RPE cells and Bruch’s membrane [2]. Advanced AMD is subdivided into exudative and non-exudative AMD. In exudative AMD, the RPE produces excessive amounts of vascular endothelial growth factor (VEGF), and this promotes the breakdown of the blood-retinal barrier and the development of choroidal neovascularization (CNV). The CNVs can penetrate Bruch’s membrane and pass into the subretinal space, and the leakage of blood from these abnormal vessels can cause an acute reduction of vision [3].

Anti-VEGF therapies are the most commonly used treatment for these eyes, and they significantly suppress the leakage from CNVs and reduce the risk of blindness [4]. While anti-VEGF therapies have improved the visual function for many patients, they require multiple injections that are expensive [5]. Considering these limitations of anti-VEGF therapies, alternative strategies to treat exudative AMD are needed.

There are some evidences that chronic intraocular inflammation might be an important mechanism for the development of exudative AMD. The evidences consist of the presence of immune cells including macrophages and microglial cells in AMD lesions, and the presence of inflammatory molecules such as vitronectin, immunoglobulin, and complement proteins in drusen [3]. In particular, the recruitment of Iba-1^+^ myeloid cells into the subretinal area has been suggested to contribute to the exacerbation of retinal degenerative diseases such as AMD and inherited retinal disease [6]. Several genetic modifications in mice, e.g., deletion of CX3CR1 and CCL2, significantly affect the microglial accumulation in the subretinal region and the formation of CNVs [7, 8]. Therefore, determining the mechanisms causing the myeloid cell-dependent inflammation in the subretinal area might lead to a new therapy for exudative AMD.

Progranulin (PGRN) is a precursor of a group of 6-kDa peptides called granulins that are commonly present in inflammatory secretions [9]. PGRN is a growth factor which is mainly found in microglial cells and neurons in the central nervous system. It plays important roles in a diverse array of biological processes such as embryonic development, cell proliferation, angiogenesis, tumorigenesis, wound repair, and inflammation [10]. Mutations in PGRN are linked to some neurodegenerative diseases including frontotemporal dementia (FTD), and to one type of lysosomal storage disease called neuronal ceroid lipofuscinosis (NCL) [11, 12]. Importantly, some studies have reported that individuals with PGRN haploinsufficiency and PGRN knockout mice (*Grn*^*−/−*^) exhibit progressive retinal degeneration. These findings indicate that PGRN might be essential for maintaining retinal homeostasis [13, 14]. In our earlier studies, we found that PGRN plays an important role in the development and maturation of the retina [15]. Moreover, PGRN deficiency affected the number of immune cells in the developing retina [16]. However, its role in age-related eye diseases is poorly understood.

Thus, the purpose of this study was to investigate the role played by PGRN in the pathology of exudative AMD.

## Methods

### Animals

Male adult C57BL/6J mice were purchased from Japan SLC (Hamamatsu, Japan). *Grn*^−/−^ mice generated by Kayasuga et al. [17] were obtained from Riken BioResource Center (Tsukuba, Japan) and were backcrossed with C57BL/6J mice. All mice were housed in an air-conditioned room maintained at 22 ± 2 °C under 12:12 h light/dark cycle. The mice had free access to a standard diet (CLEA Japan) and tap water. The number of mice used for each experiment is indicated in the figure legends.

### Laser-induced choroidal neovascularization model

The mice were anesthetized by an intramuscular injection of a mixture of ketamine (43.8 mg/kg; Daiichi-Sankyo) and xylazine (2.5 mg/kg; Bayer Healthcare). The pupils were dilated with 0.5% tropicamide (Santen Pharmaceutical), and laser photocoagulation (647 nm, 120 mW, 100 ms, 50 μm; MC500, NIDEC) was performed on the right eye of each animal on day 0. Six laser spots were made around the optic disc. The endpoint of the laser burn was the appearance of a cavitation bubble which was correlated with the disruption of Bruch’s membrane. The following laser spots were excluded from the studies: (1) Laser spots with bleeding immediately after photocoagulation, (2) Laser spots with bleeding scar or connections between other spots at the endpoint.

### Immunostaining of ocular sections

For immunostaining the ocular tissues, the eyes were enucleated and fixed in 4% paraformaldehyde for at least 24 h at 4 °C, and then immersed in 25% sucrose in 0.01 M phosphate-buffered sarin (PBS) for 2 days. The eyes were then embedded in optimal cutting temperature (OCT) compound (Sakura Finetek Japan) and immediately frozen with liquid nitrogen. Ten-micrometer sections were cut with a cryostat, and the sections were mounted on glass slides (MAS COAT; Matsunami Glass).

The retinal sections were blocked in non-immune horse serum (Vector Labs) for 1 h and then incubated with the primary antibody at 4 °C overnight. The next morning the sections were covered with a secondary antibody for 1 h and then counterstained with Hoechst 33342 (1:1000; Invitrogen, catalog H3570) for 15 min.

The following antibodies were used; sheep anti-mPGRN (1:100; R&D Systems, catalog AF2557), rabbit anti-Iba1 (1:200; FUJIFILM Wako, catalog 019-19741), rat anti-CD68 (1:200; Bio-Rad, catalog MCA1957GA), rat anti-LAMP1 (1:500; abcam, catalog ab25245), goat anti-cathepsin D (1:500; R&D systems, catalog AF1029), Alexa Fluor^®^ 647 donkey anti-sheep IgG (1:1000; Invitrogen, catalog A21448), Alexa Fluor^®^ 546 donkey anti-rabbit IgG (1:1000; Invitrogen, catalog A10040), and Alexa Fluor^®^ 488 donkey anti-rat IgG (1:1000; Invitrogen, catalog A21208). The stained sections were examined and photographed with a confocal microscope (FLUOVIEW FV10i; Olympus).

### Fundus fluorescein angiography

Two weeks after the photocoagulation (day 14), the mice were anesthetized by an intramuscular injection of ketamine and xylazine. After dilation of the pupils and intravenous administration of 0.1 mL of a tenfold saline dilution of 10% fluorescein (Alcon Japan), fundus fluorescein angiography (FFA) was performed with a Micron IV Retinal Imaging Microscope (Phoenix Research Laboratories). The grade of leakage was assigned as described below: 1, “no leakage,” faint hyperfluorescence or mottled fluorescence; 2, “questionable leakage,” hyperfluorescent lesion without a progressive increase in intensity or size; 3, “leaky,” hyperfluorescence increasing in intensity but not in size; 4, “pathologically significant leakage,” hyperfluorescence increasing in both intensity and size.

### Quantifications of choroidal neovascularization

After confirming that the mice were under deep anesthesia, mice were administrated 0.5 mL PBS containing 20 mg/mL fluorescein-conjugated dextran (MW ≈ 2000 kDa, Sigma-Aldrich) through the tail vein. Five minutes after administration of fluorescein-conjugated dextran, their eyes were enucleated and fixed in 4% paraformaldehyde for 12 h. The cornea and lens were removed while viewing the eye under a microscope, and the retinas were carefully peeled from the RPE-choroid-sclera complex. The RPE-choroid-sclera complexes were flat-mounted and covered with a micro cover glass (Matsunami Glass) after a few drops of fluoromount (DBS Diagnostic Biosystems) were placed on the microscope slide. The slides were photographed with BZ-X710 (Keyence) for the overall picture and FLUOVIEW FV10i (Olympus) for the laser spots. The areas of the CNV were measured using the ImageJ analysis software (National Institutes of Health).

### Immunostaining of choroidal flat mounts

The eyes for immunostaining were enucleated 7 or 14 days after photocoagulation and then fixed in 4% paraformaldehyde for 12 h. The RPE-choroid-sclera complex was separated from the retina, isolated, and blocked in non-immune horse serum (Vector Labs) for 1 h, and then incubated with the primary antibody at 4 °C overnight. Then, the RPE-choroid-sclera complex was stained with a secondary antibody for 1 h, and flat-mounted on the slide. The slides were viewed and photographed for an overall picture and FLUOVIEW FV10i (Olympus) for the laser spots. The intensity of CD68 and VEGF-A in the CNV lesion was determined by the ImageJ analysis software (National Institutes of Health). The accumulation of Iba-1^+^ myeloid cells around the CNV was counted in Iba-1-stained whole RPE-choroidal flat mounts viewed from the RPE side. The primary antibodies used were as follows: rabbit anti-Iba1 (1:200; FUJIFILM Wako, catalog 019-19741), sheep anti-mPGRN (1:200; R&D systems, catalog AF2557), rabbit anti-VEGF-A (1:200; Merck Millipore, catalog PC315), rat anti-CD68 (1:200; Bio-Rad, catalog MCA1957GA), Alexa Fluor^®^ 647 donkey anti-sheep IgG (1:1000; Invitrogen, catalog A21448), Alexa Fluor^®^ 647 donkey anti-rat IgG (1:1000; Jackson ImmunoResearch, catalog 712-605-153), and Alexa Fluor^®^ 546 donkey anti-rabbit IgG (1:1000; Invitrogen, catalog A10040).

### Cell cultures

A mouse macrophage cell line (RAW264.7) was obtained from the American Type Culture Collection (Manassas, USA). The RAW264.7 cells were grown in Dulbecco’s modified Eagle’s medium (DMEM; Nacalai Tesque, catalog 08456-36) containing 10% fetal bovine serum (FBS) in a humidified atmosphere of 95% air and 5% CO_2_ at 37 °C. The cells were passaged by trypsinization every 2 to 3 days, and subconfluent monolayers of RAW264.7 cells from passages 10 to 16 were used in the experiments.

### Transfection by small interfering RNA and cell treatment

To suppress the expression of PGRN in the RAW264.7 cells, three small interfering RNA (siRNA) sequences targeting *Grn* were synthesized by Invitrogen (catalog 1320001). The siRNAs (20 nmol) were transfected into RAW264.7 cells for 48 h with Lipofectamine^®^ RNAiMAX Reagent (Invitrogen, catalog 13778-150). The sequences of the three siRNAs for PGRN were:

siRNA-a, 5′-CCAUGAUAACCAGACCUGUAAA-3′,

siRNA-b, 5′-GGAACCAAGUGUUUGCGAAAGAAGA-3′, and

siRNA-c, 5′-GGACCUGUGAGAAGGAUGUCGAUUU-3′.

To induce an abnormal activation of macrophages, the cell cultures were placed under hypoxic conditions. The RAW264.7 cells were incubated in 1% FBS serum-containing DMEM in an oxygen-free incubator (94% N_2_, 5% CO_2_, 1% O_2_) for 12 h. Control cells were incubated under normoxic conditions. After the hypoxic incubation, cell viability assay, western blotting, and immunostaining were performed. The cellular viability was determined with the Cell Counting Kit 8 (Dojindo Molecular Technologies, catalog 343-07623). For this, the cells were incubated with 10% of 2-(2-methoxy-4-nitorphenyl)-3-(4-nitrophenyl)-5-(2,4-disulfophenyl)-2H-tetrazolium, monosodium salt for 1 h at 37 °C. The optical density at 450 nm was measured with a microplate reader (Varioskan Flash 2.4; Thermo Fisher Scientific).

### Primary macrophage culture

Peritoneal macrophages from *Grn*^*+/+*^ and *Grn*^*−/−*^ mice (10–12 weeks) were collected by lavage of the peritoneal cavity with 5 mL of sterile FBS-free DMEM (Nacalai Tesque, catalog 08456-36). The cells were centrifuged and suspended in FBS-free DMEM, and the obtained cells were seeded in 24- or 96-well plates at densities of 5 × 10^4^ or 1 × 10^4^ cells/well, respectively. Thirty minutes after seeding, any unattached cells were washed out with FBS-free medium, and then, the cells were grown in DMEM containing 10% FBS in a humidified atmosphere of 95% air and 5% CO_2_ at 37 °C. To induce the abnormal activation of macrophages, the cells seeded in 24 well plates were incubated in an oxygen-free incubator (94% N_2_, 5% CO_2_, 1% O_2_) for 6 h.

### In vitro immunostaining

The RAW264.7 cells were fixed in 2.67% paraformaldehyde at room temperature for 30 min. The cells were then incubated with 0.2% Triton X-100 (Bio-Rad, catalog 1610407) in PBS for 30 min and blocked with 1% bovine serum albumin (Nacalai tesque, catalog 01863-06) for 1 h. The cells were incubated with the primary antibodies overnight at 4 °C and then incubated with secondary antibodies and Hoechst 33342 (1:1000; Invitrogen) for 1 h. The following antibodies were used; rat anti-CD68 (1:200; Bio-Rad, catalog MCA1957GA), rabbit anti-iNOS (1:200; Cell signaling technology, catalog 13120), Alexa Fluor^®^ 488 donkey anti-rat IgG (1:1000; Invitrogen, catalog A21208), and Alexa Fluor^®^ 546 donkey anti-rabbit IgG (1:1000; Invitrogen, catalog A10040). The images were taken with a FLUOVIEW FV10i (Olympus) fluorescent microscope.

### Western blot analysis

For the western blot analyses, the eyes were enucleated after cervical dislocation, and the retinas and RPE-choroid-sclera complexes were isolated and rapidly frozen in liquid nitrogen. To extract the proteins, the tissue was homogenized in RIPA buffer (Sigma-Aldrich, catalog R0278) containing a protease inhibitor and a phosphatase inhibitor cocktail with a homogenizer (Microtec Co., Ltd.). In addition, the RAW264.7 cells and peritoneal macrophages in 24 well plates were lysed in the same buffer. The lysate was centrifuged at 12,000 g for 20 min, and the protein concentration was measured by comparison with known concentrations of BSA with a bicinchoninic acid protein assay kit (Pierce Chemical, catalog 23225). The sample buffer with 3-Mercapto-1,2-propanediol (FUJIFILM wako, catalog 196-16142) was added (sample:sample buffer = 3:1) and boiled for 5 min.

The protein samples were separated on 5–20% and 15% SDS-PAGE gels and then transferred onto a polyvinylidene difluoride membrane (Immobilon-P; Millipore, catalog IPVH00010). The following primary antibodies were used: sheep anti-mPGRN (1:200; R&D systems, catalog AF2557), rabbit anti-VEGF-A (1:200; Merck Millipore, Catalog PC315), rabbit anti-IL-1β (1:200; Abcam, catalog ab9722), mouse anti-C3 (1:200; Santa Cruz, catalog sc-28294), rabbit anti-TNF-α (1:1000; Cell Signaling Technology, catalog 11948), rat anti-CCL2 (1:200; Santa Cruz, catalog sc-52701), mouse anti-sortilin (1:200; Santa Cruz, catalog sc-376561), rat anti-LAMP1 (1:500; Abcam, catalog ab25245), Goat anti-cathepsin D (1:500; R&D systems, catalog AF1029), and mouse anti-β-actin (1:2000; Sigma-Aldrich, catalog A2228). After exposure to the primary antibodies for at least 12 h, the membranes were incubated with horseradish peroxidase (HRP)-conjugated rabbit anti-sheep IgG, goat anti-rabbit IgG (1:2000; Thermo Scientific), goat anti-rat IgG (1:2000; Thermo Scientific), or goat anti-mouse IgG (1:2000; Thermo Scientific), rabbit anti-goat IgG (1:2000; Thermo Scientific) for 1 h at room temperature. The immunoreactive bands were made visible with ImmunoStar LD (FUJIFILM Wako, catalog 290-69904) and then measured with the Amersham Imager 680 blot and gel imager (GE Healthcare Life Sciences).

### Lysotracker staining

LysoTracker Red DND-99 (LTR; Invitrogen, catalog L7528) was dissolved in PBS (50 nM) and stored at 4 °C. An aliquot of the stock solutions of the dye was added to the culture media. Prior to the measurements, cells were incubated with the dye for 15 min at 37 °C.

### Statistical analyses

The data are expressed as the means ± SEMs of at least 3 independent mice, eyes, or wells. The normality of each group was tested by the Shapiro-Wilk test. Two data sets were compared using two-tailed Welch’s *t*-test and Dunn-Bonferroni post hoc method following significant Kruskal-Wallis tests. Multiple comparisons were performed using the Dunn-Bonferroni post hoc method following significant Kruskal-Wallis tests and one-way ANOVA followed by Tukey’s or Dunnett’s post hoc test. A *P* value of < 0.05 was considered statistically significant. All statistical analyses were performed using SPSS (version 24.0.0.0; IBM, Armonk, NY, USA) software.

## Results

### Expression level of PGRN in eyes of CNV mouse model

**Fig. 1:**
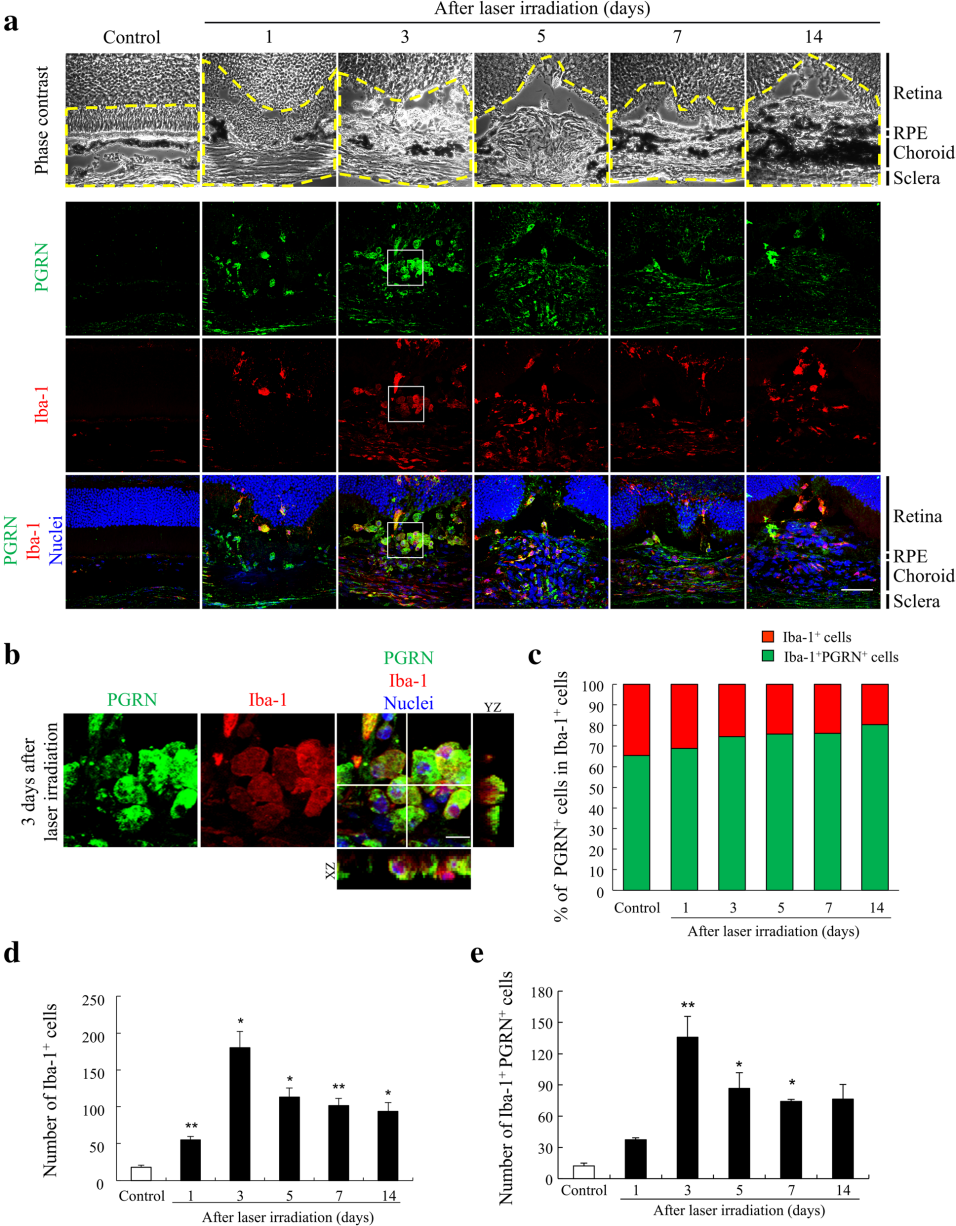
Expression level of progranulin in eyes of a choroidal neovascularization (CNV) model mouse. **a** Immunofluorescence staining of the laser-injured eye of C57BL/6J mice (8 weeks old) at 0, 1, 3, 5, 7, and 14 days after the photocoagulation with anti-PGRN (green) and anti-Iba-1 (red) antibodies. Nuclei were stained with Hoechst 33342 (blue). Phase contrast images are also shown. The yellow dotted line shows the area that was counted. Scale bar: 50 μm. **b** Enlarged images at 3 days after laser coagulation. Scale bar: 10 μm. **c** Graph of the ratio of PGRN^+^ cells in Iba-1^+^ cells around CNVs. Data are presented as percentages. Expression of PGRN was observed in 65–80% of Iba-1^+^ cells in CNV lesions. **d**, **e** Quantitative analysis of the number of Iba-1^+^ cells and PGRN^+^Iba-1^+^ cells in the subretinal area at 0, 1, 3, 5, 7, and 14 days after photocoagulation. Data are the means ± SEMs, (*n* = 5). ^*^P <0.05, ^**^P <0.01 vs. Control (**d**: one-way ANOVA followed by Dunnett’s test, **e**: Dunn-Bonferroni post hoc method following significant Kruskal-Wallis tests)

To examine the pathological role of PGRN in the eye, we performed laser photocoagulation to induce the development of CNVs [18] in adult C57BL/6J mice. We examined the expression and location of PGRN and Iba-1^+^ myeloid cells around the photocoagulated lesion by immunofluorescence staining of retinal cross sections. The expression level of PGRN around the photocoagulated choroid was significantly higher than in normal eyes (Fig. 1A). At the same time, the Iba-1^+^ myeloid cells were found to be accumulated in the laser-irradiated sites, and PGRN was seen to be located in these cells (Fig. 1A and B). Expression of PGRN was observed in 65-80% of iba-1^+^ cells in CNV lesions (Fig. 1C). The peak of the accumulation of Iba-1^+^ cells and PGRN^+^Iba-1^+^ cells in the subretinal area was 3 days after photocoagulation, and these cells remained at the lesion site even 14 days after the laser coagulation (Fig. 1D and E).

We also determined the level of expression of PGRN in the retina and RPE-choroid-sclera complex by western blotting. While the expression level of PGRN in laser-irradiated retina did not change significantly, a significant increase in PGRN was confirmed in the RPE-choroid-sclera complex at 3 and 5 days after the laser coagulation (Supplemental Figures 1A, B).

### PGRN deficiency exacerbates vascular permeability from CNV

**Fig. 2:**
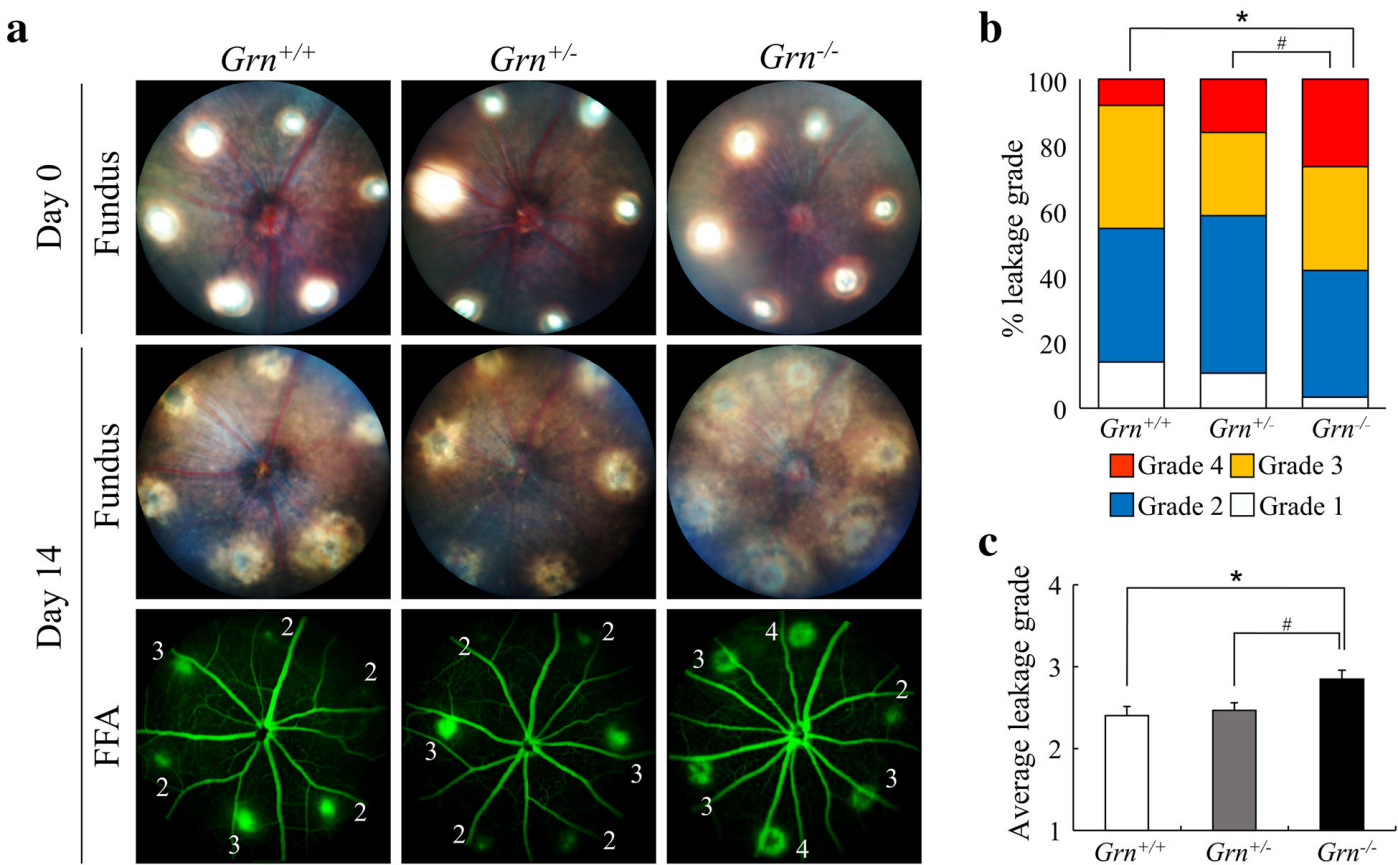
Increased vascular leakage from CNV in progranulin knockout mice. **a** Representative photographs of ocular fundus from *Grn*^*+/+*^, *Grn*^*+/−*^, and *Grn*^*−/−*^ mice (9–12 weeks) immediately after laser coagulation. Angiographic images at 14 days after photocoagulation. **b**. Graph of the FFA grade scores of each laser spot (*Grn*^*+/+*^, 64 laser spots; *Grn*^*+/−*^, 75 laser spots; and *Grn*^*−/−*^, 57 laser spots). The comparison of each group was performed using the laser spots of all grades. Data are presented as percentages. ^*^P <0.05 vs. *Grn*^*+/+*^, ^#^*P* <0.05 vs. *Grn*^*+/−*^ (Dunn-Bonferroni post hoc method following significant Kruskal-Wallis tests). **c**. Graph of the average leakage grade. (*Grn*^*+/+*^, n = 11; *Grn*^*+/*^^−^, n = 13; and *Grn*^*−/−*^, n = 10). Data are the means ± SEMs. ^*^P <0.05, vs. *Grn*^*+/+*^; ^#^*P* <0.05, vs. *Grn*^*+/−*^ (one-way ANOVA followed by Tukey’s test)

To examine the effects of PGRN deficiency, laser photocoagulation was performed around the optic nerve head in *Grn* WT (*Grn*^*+/+*^), *Grn* heterozygous (*Grn*^*+/−*^), and *Grn* deficient (*Grn*^*−/−*^) C57BL/6J mice. Laser burns were identified in the fundus images immediately after the laser photocoagulation and 14 days after the photocoagulation. These images indicated clear differences in the size of the laser-induced scars between *Grn*^*−/−*^, *Grn*^*+/+*^, and *Grn*^*+/−*^ mice at 14 days post-photocoagulation (Fig. 2A). The vascular permeability was determined by fluorescein fundus angiography (FFA). Our results showed that there was no difference in fluorescein leakage between *Grn*^*+/+*^ and *Grn*^*+/−*^ mice; the FFA disclosed increased fluorescein leafage in *Grn*^*−/−*^ mice compared with *Grn*^*+/+*^ and *Grn*^*+/−*^ mice (Fig. 2A). The distribution and proportion of the lesion grades in *Grn*^*−/−*^ mice significantly increased from those in *Grn*^*+/+*^ and *Grn*^*+/−*^ mice (Fig. 2B). On average, the leakage grade was significantly higher in *Grn*^*−/−*^ mice compared with *Grn*^*+/+*^ and *Grn*^*+/−*^ mice (Fig. 2C).

### PGRN deficiency increases CNV area and number of infiltrating Iba-1^+^ myeloid cells around CNVs

**Fig. 3:**
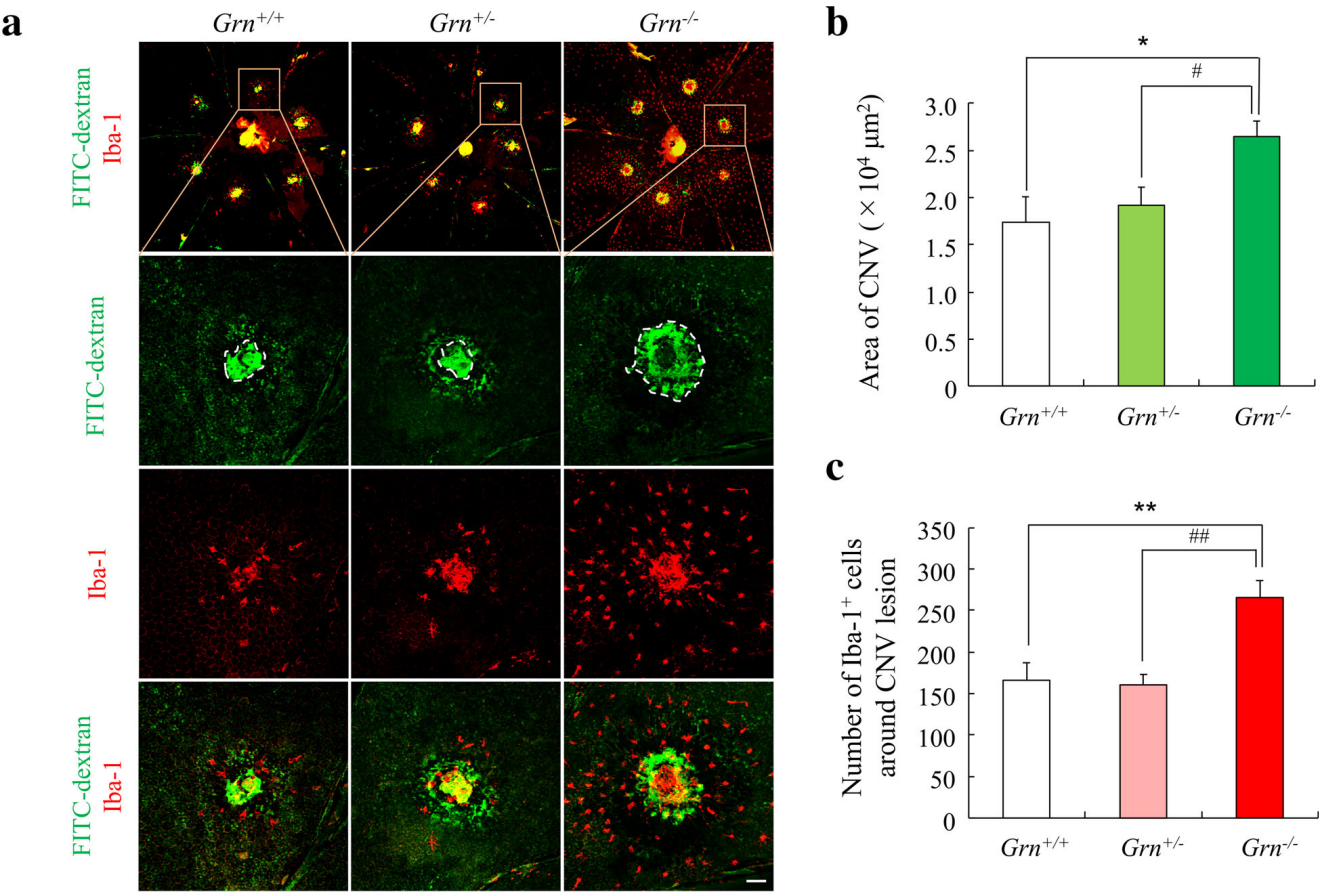
Larger size laser-induced CNV lesion and higher number of Iba-1^+^ cells in the progranulin knockout mice. **a** Representative microscopic images of FITC-dextran angiogram and immunostaining of Iba-1 of RPE-choroid flat mounts from *Grn*^*+/+*^, *Grn*^*+/−*^, and *Grn*^*−/−*^ mice (9-12 weeks). White dotted line shows CNV area. Scale bars: 50 μm. **b**. Quantification of the mean size of the CNV areas. **c** Quantification of the number of Iba-1^+^ cells around the CNVs (*Grn*^*+/+*^, n = 9; *Grn*^*+/−*^, n = 14; and *Grn*^*−/−*^, n = 10). Data are the means ± SEMs. ^*^P <0.05, ^**^P <0.01 vs. *Grn*^*+/+*^; ^#^*P* <0.05, ^##^*P* <0.01 vs. *Grn*^*+/−*^ (**b**: Dunn-Bonferroni post hoc method following significant Kruskal-Wallis tests, **c**: one-way ANOVA followed by Tukey’s test)

The size of the CNV area was also determined by FITC-dextran angiography at 14 days after the laser coagulation. Consistent with the FFA grades, the mean size of the CNV lesions was significantly larger in *Grn*^*−/−*^ mice than in *Grn*^*+/+*^ and *Grn*^*+/−*^ mice. However, there was no significant difference in the size of the CNV between *Grn*^*+/+*^ and *Grn*^*+/−*^ mice (Fig. 3A, B).

Infiltrating retinal microglia and systemic macrophages play an important role in the development of a CNV [19]. To determine the effects of the infiltration of Iba-1^+^ myeloid cells around the CNV, immunostaining with anti-Iba-1 antibody was performed on whole RPE-choroidal flat mounts. The results showed that *Grn*^*−/−*^ mice had significantly more Iba-1^+^ cells around the CNV than *Grn*^*+/+*^ and *Grn*^*+/−*^ mice (Fig. 3A, C).

### PGRN deficient macrophages have proangiogenic phenotype

**Fig. 4:**
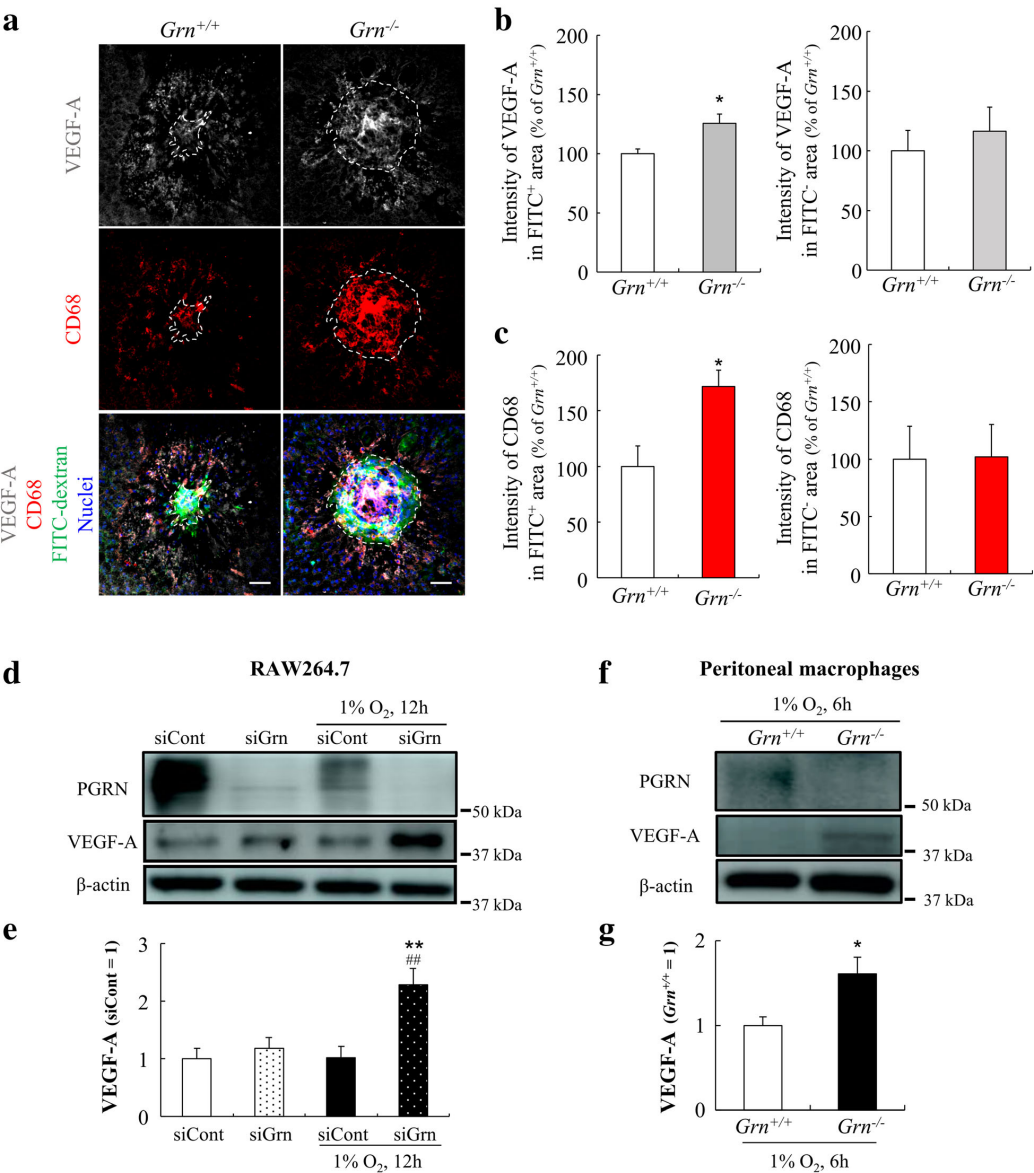
Proangiogenic phenotype of progranulin deficient macrophages. **a** Immunofluorescent staining of laser-irradiated RPE-choroid complex from *Grn*^*+/+*^ and *Grn*^*−/−*^ mice (9–12 weeks) with anti-VEGF-A (white) and anti-CD68 (red) antibodies. Nuclei and CNV are stained with Hoechst 33342 (blue) and FITC-dextran (green). The white dotted line shows the CNV area. Scale bars, 50 μm. **b**, **c** Quantification of mean gray value of VEGF-A (**b**) and CD68 (**c**) in FITC^+^ and FITC^-^ area (*Grn*^*+/+*^, n = 3; and *Grn*^*−/−*^, n = 4). Data are the means ± SEMs. ^*^P < 0.05 vs. *Grn*^*+/+*^ (Welch’s *t*-test). **d**, **e** PGRN and VEGF-A expression levels in RAW264.7 cells after transfection by siControl or siGrn#1 and exposure to hypoxia. Data are the means ± SEMs (n = 4). ^*^P <0.05; ^**^P <0.01 vs. siControl; ^#^*P* <0.05; ^##^*P* <0.01 vs. siControl + Hypoxia (one-way ANOVA followed by Tukey’s tests). **f**, **g** PGRN and VEGF-A expression levels in *Grn*^*+/+*^ or *Grn*^*−/−*^ mice-derived peritoneal macrophages after exposure to hypoxia (n = 4). Data are the means ± SEMs. ^*^P < 0.05 vs. *Grn*^*+/+*^ (Welch’s *t*-test)

Seven days after the laser coagulation, the expressions of VEGF-A and CD68 were determined by immunofluorescence staining. CD68 was expressed predominantly on the lysosomal membranes of Iba-1^+^ myeloid cells [20]. We also confirmed that CD68 is expressed in the cytoplasm of Iba-1-positive cells with IHC and ICC (Supplemental Fig. 3A, B). Therefore, we used CD68 as a myeloid cell marker in this experiment. The fluorescence intensity of VEGF-A in the FITC^+^ area of *Grn*^*−/−*^ mice was higher than that in *Grn*^*+/+*^ mice which was consistent with the intensity level of CD68^+^ myeloid cells while the intensity of both VEGF-A and CD68 in the FITC^-^ area did not significantly changed between *Grn*^*+/+*^ and *Grn*^*−/−*^ mice (Fig. 4A–C).

To examine the role played by the PGRN in macrophages, we transfected small interfering RNA (siRNA) sequences targeting the PGRN in RAW264.7 cells, a mouse macrophage cell line, to silence the expression of PGRN in the cells. We first examined whether the PGRN could be silenced by the three different siRNAs targeting granulin (siGrn). Our results showed that the expression level of PGRN in RAW264.7 cells after 24–48 h transfection was higher in the scrambled siRNA (siControl) group than siGrns treated groups (Supplemental Figure 4A, B).

Earlier studies have reported that hypoxia is one of the key inducers of CNV formation [21]. To mimic the environment around the CNV *in vitro*, PGRN silenced macrophages were incubated under hypoxic condition (1% O_2_) for 12 h to induce abnormal activation. Compared to siControl treated cells, VEGF-A was upregulated in the siGrn cells under hypoxic conditions (Fig. 4D, E). Moreover, we evaluated the expression level of VEGF-A from *Grn*^*+/+*^ or *Grn*^*−/−*^ mice-derived peritoneal macrophages. These cells were incubated under hypoxic condition for 6 h. The expression level of VEGF-A in *Grn*^*−/−*^ mice-derived macrophages significantly increased than in *Grn*^*+/+*^ mice-derived cells (Fig. 4F, G).

### SiGrn treatment of macrophage cell line upregulates expression of inflammatory cytokines

**Fig. 5:**
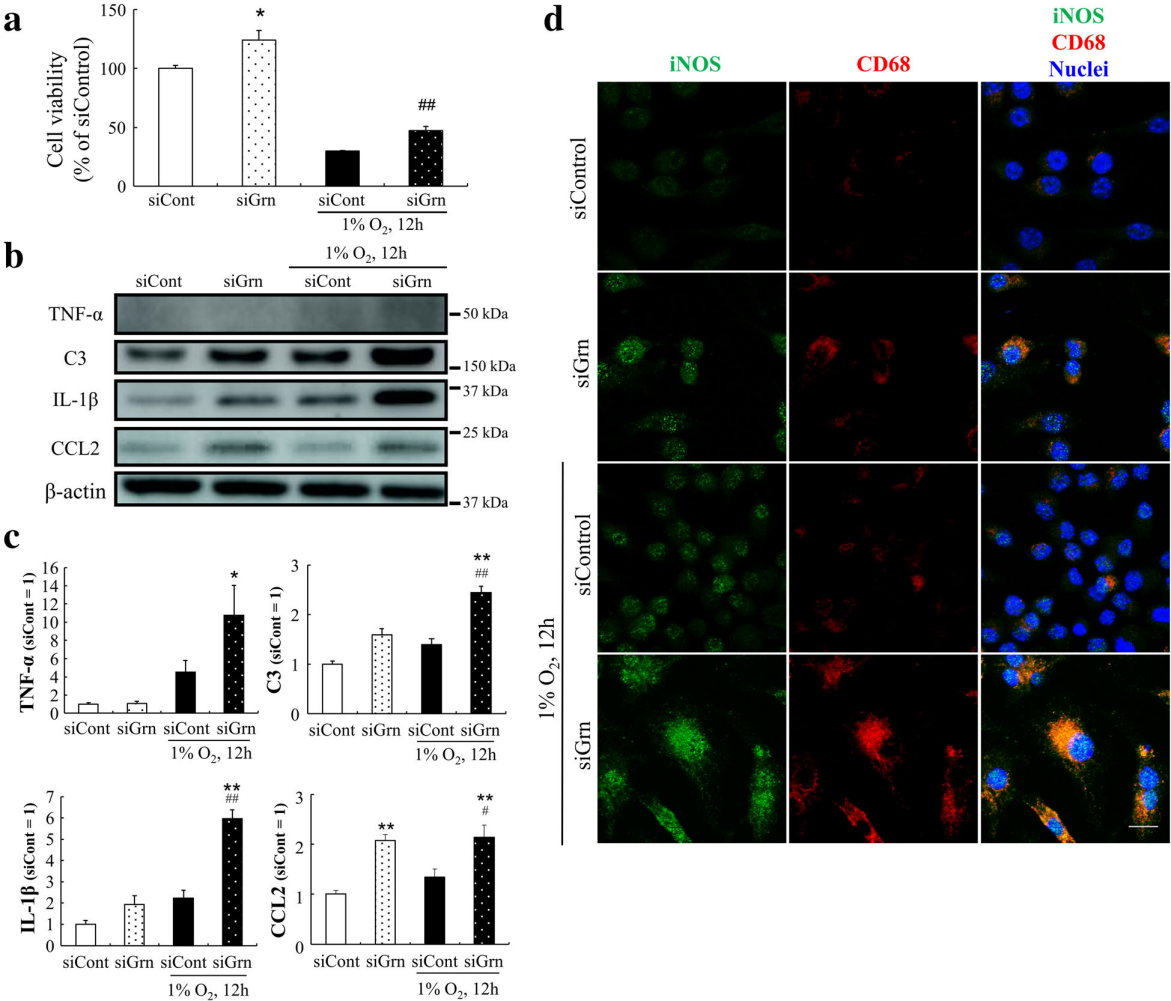
Higher levels of proinflammatory cytokines in progranulin silenced RAW264.7 cells. **a** Cell viability of RAW264.7 cells after transfection in siControl or siGrn mice and exposure to hypoxia. Data are the means ± SEMs (n = 6). ^*^P <0.05 vs. siControl; ^##^*P* <0.01 vs. siControl + Hypoxia (Welch’s *t*-test). **b** Representative western blots showing immunoreactivity against TNF-α, C3, IL-1β, CCL2, and β-actin. **c** Quantitative analysis of expression level of TNF-α, C3, IL-1β, and CCL2 in RAW264.7 cells. Data are the means ± SEMs (n = 4). ^*^P <0.05; ^**^P <0.01 vs. siControl; ^#^*P* <0.05, ^##^*P* <0.01 vs. siControl + Hypoxia (TNF-α and C3: Dunn-Bonferroni post hoc method following a significant Kruskal-Wallis tests, IL-1β and CCL2: one-way ANOVA followed by Tukey’s tests). **d** Immunofluorescence staining of RAW264.7 cells with anti-iNOS (green) and anti-CD68 (red) antibodies. Nuclei were stained with Hoechst 33342 (blue). Scale bar: 10 μm

To examine the effects of PGRN silencing on the activation of macrophages, we determined the cell viability and expression levels of inflammatory cytokines. The cell viability of siGrn-treated RAW264.7 cells was higher than that of the siControl-treated group in both the hypoxia and normoxia groups (Fig. 5A). When siGrn-exposed RAW264.7 cells were incubated under hypoxic conditions, several proinflammatory cytokines, viz., tumor necrosis factor-α (TNF-α), complement component 3 (C3), interleukin-1β (IL-1β), and C–C motif chemokine ligand 2 (CCL2) were increased in the siGrn- and hypoxia-treated RAW264.7 cells (Fig. 5B, C). Moreover, the expression of inducible nitric oxide synthase (iNOS) was increased in the cells which is one of the markers of activated myeloid cells (Fig. 5D).

### PGRN-deficient myeloid cells show lysosomal abnormality

**Fig. 6:**
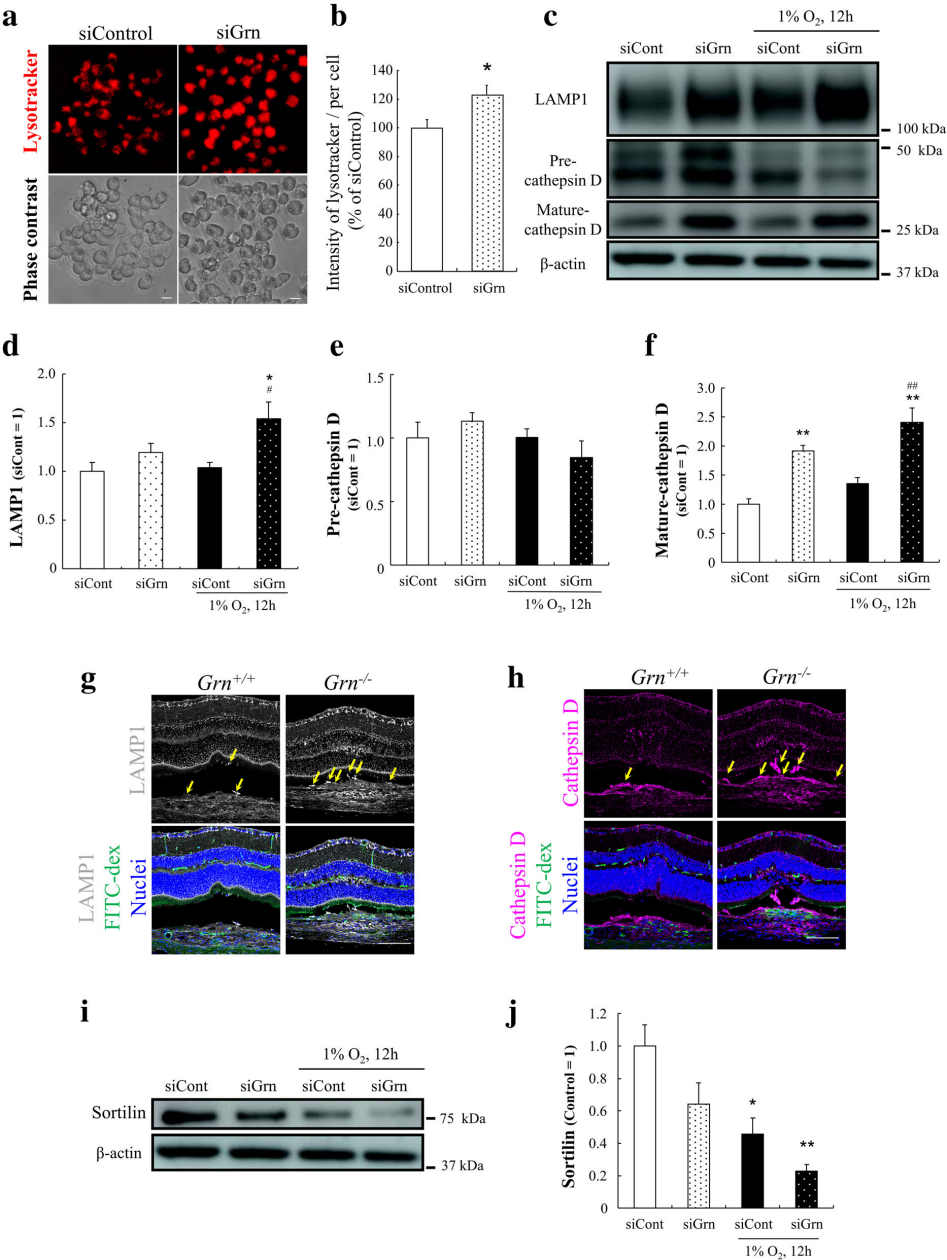
Lysosomal abnormality in PGRN-deficient macrophages. **a** Representative images of LysoTracker Red DND-99 staining (red) and phase contrast images of RAW264.7 cells after transfection by siControl or siGrn. Scale bar: 10 μm. **b** Quantitative analysis of the fluorescence intensity of LysoTracker^+^ cells. Data are the means ± SEM (n = 6). ^*^P <0.05 vs. siControl (Welch’s *t*-test). **c** Representative images of western blots showing immunoreactivity against LAMP1, cathepsin D, and β-actin. **d**–**f** Quantitative analysis of expression level of LAMP1 (**d**), pre-cathepsin D (**e**), and mature-cathepsin D (**f**) in RAW264.7 cells after transfection of siControl or siGrn and exposure to hypoxia (1% O_2_, 12 h). Data are the means ± SEMs (n = 4). ^*^P <0.05; ^**^P <0.01 vs. siControl; ^#^*P* <0.05; ^##^*P* <0.01 vs. siControl + Hypoxia (one-way ANOVA followed by Tukey’s tests). **g**, **h** immunohistochemistry on the laser-injured eye of *Grn*^*+/+*^ and *Grn*^*−/−*^ mice 7 days after the photocoagulation with anti-LAMP1 (white) and anti-cathepsin D (magenta) antibodies. Nuclei were stained with Hoechst 33342 (blue) and the CNV regions were visualized by FITC-dextran (green). Yellow arrows show LAMP1^+^ or cathepsin D^+^ cells infiltrated into the CNV area. Scale bar: 100 μm. **i**, **j** Level of expression of sortilin in RAW264.7 cells after transfection by siControl or siGrn and exposure to hypoxia. Data are the means ± SEMs (n = 4). ^*^P <0.05; ^**^P <0.01 vs. siControl (one-way ANOVA followed by Tukey’s tests)

Lysosomal staining by a red fluorescent dye for labeling and tracking the acidic organelles (LysoTracker Red DND-99) was performed to evaluate the amount of lysosome in the PGRN-silenced macrophages. The fluorescence intensity of LysoTracker in the PGRN-silenced macrophages was significantly higher than that in the control cells (Fig. 6A, B). In addition, the expression level of lysosomal-associated membrane protein 1 (LAMP1) was higher in the siGrn- and hypoxia-treated RAW264.7 cells compared to siControl and normoxia-exposed cells (Fig. 6C, D). Cathepsin D is a lysosomal protease which requires cleavage steps from an inactive precursor (pre-cathepsin D) to the mature state (mature-cathepsin D). In siGrn- and hypoxia-treated cells, the level of mature-cathepsin D was higher while the level of pre-cathepsin D was lower (Fig. 6C, E, F). Similar results were obtained from experiments with *Grn*^*+/+*^ and *Grn*^*−/−*^ mouse-derived peritoneal macrophages (Supplemental fig. 5). The expression levels of LAMP1 and cathepsin D were also observed on retinal cross sections of *Grn*^*+/+*^ and *Grn*^*−/−*^ mice. Seven days after the laser coagulation, the expression of these proteins in the CNV lesion of *Grn*^*−/−*^ mice increased than that in *Grn*^*+/+*^ mice. Moreover, the number of infiltrated cells with these lysosomal proteins into CNV lesion of *Grn*^*−/−*^ mice is higher than the number of these cells in *Grn*^*+/+*^ mice (Fig. 6G, H).

Sortilin is a transmembrane protein of the VPS10 family and is known to be one of the receptors for PGRN, and it mediates the delivery of PGRN into lysosomes [22]. The expression level of sortilin in siGrn-treated RAW264.7 cells was downregulated. In hypoxia-exposed cells, the lower levels of sortilin were greater than those in the normoxia-exposed group (Fig. 6I, J).

## Discussion

The clinical relevance of PGRN in several autoimmune and chronic diseases, including rheumatoid arthritis, inflammatory bowel disease, diabetes mellitus, and atherosclerosis, has been reported [23–26]. The loss of PGRN function can lead to the onset of several neurodegenerative diseases, e.g., FTLD and NCL, and it is accompanied by abnormal microglial activation [27]. Abnormally activated myeloid cells cause inflammatory conditions in the central nervous systems and lead to neuronal death. However, its role in chronic eye diseases is poorly understood.

Our findings demonstrated that Iba-1^+^ myeloid cells including microglia and peripheral macrophages migrate and accumulate around the CNV sites and the expression level of PGRN increase after laser photocoagulation in wild-type mice. The pattern of the absolute numbers of Iba1^+^PGRN^+^ cells suggests that there is an increase in PGRN^+^ cells over time. However, the numbers of Iba1^+^ cells follow the same kinetic as the Iba1^+^PGRN^+^ cells while the relative percentages do not significantly change at all over time (Fig. 1). It indicates that once the myeloid cells are accumulated in the subretinal area, they express and maintain the PRGN expression over the investigated time span. These data suggest that a significant increase in PGRN in the RPE-choroid-sclera after photocoagulation mainly caused by Iba-1^+^ cells accumulated in CNV lesions. On the other hand, PGRN was not expressed at the laser-irradiated sites in *Grn*^*−/−*^ mice (Supplemental Figure 2). Regarding recruited Iba1^+^ cell populations into CNV lesion and the surrounding that, a recent study concluded that retinal microglia constitute a major cell population in the diseased retina and RPE-choroid complex with lower numbers of recruited monocytes [28]. This report indicates that the majority cell population which contributes to the secretion of PGRN in CNV lesion is retinal microglia rather than peripheral macrophages.

It has been reported that PGRN promotes angiogenesis by promoting the growth and migration of the vascular endothelial cells in wound healing and tumor genesis [29, 30]. In addition, it is also reported that PGRN could act as a chemoattractant to recruit microglia in the brain [31]. Therefore, we originally expected that the abundant PGRN secreted from infiltrating Iba-1^+^ myeloid cells at the lesion would promote the growth of CNVs and the accumulation of myeloid cells, resulting in exacerbation of the pathological condition. However, our results showed that the size of the CNV was significantly larger in *Grn*^*−/−*^ mice than in *Grn*^*+/+*^ and *Grn*^*+/−*^ mice. Moreover, the number of ocular infiltrating Iba-1^+^ myeloid cells around the CNVs was higher in *Grn*^*−/−*^ mice than that in *Grn*^*+/+*^ and *Grn*^*+/−*^ mice (Fig. 3). This difference might be due to the phenotypic changes of the Iba-1^+^ myeloid cells associated with the PGRN deficiency. In various pathological models, the number of Iba-1^+^ myeloid cells migrating to the lesion is higher in PGRN-deficient mice than in wild type mice [32, 33]. According to our results, PGRN might play a role in regulating the infiltration of Iba-1^+^ myeloid cells into CNVs.

Our results also showed that myeloid cells in the laser-induced injury site expressed VEGF-A (Fig. 4). This indicated that myeloid cells in *Grn*^*−/−*^ mice may have proangiogenic properties in the CNV lesions. VEGF-A regulates angiogenesis, enhances vascular permeability, and enhances the formation of choroidal neovascularization. In PGRN-deficient mice, the increased accumulation of myeloid cells and subsequent secretion of VEGF-A could be responsible for the development of the CNVs. Moreover, the increased VEGF-A expression from myeloid cells could also contribute to an increase in vascular permeability from the CNVs. Our results showed that the vascular permeability in *Grn*^*−/−*^ mice was significantly higher than that in the *Grn*^*+/+*^ and *Grn*^*+/−*^ mice (Fig. 2). These results suggest that the level of expression of proangiogenic factor VEGF-A from Iba-1^+^ myeloid cells was controlled by PGRN in the inflammatory lesion. Earlier studies have provided strong evidence that activated myeloid cells play a major role in the exacerbation of exudative AMD, e.g., myeloid cells in the CNV lesions express VEGF-A in patients with exudative AMD, and pharmacological inhibition of myeloid cell infiltration into the subretinal space significantly reduced the area of the CNV in the laser-induced CNV model [19, 34–36]. On the contrary, some reports concluded that monocyte-derived VEGF-A is not necessary for CNV formation [37, 38]. In addition, it has been reported that Iba-1^+^ myeloid cells indirectly contribute to the exacerbation of CNV through upregulation of VEGF-A expression in Müller cells [39]. These reports support our results in this study. At least, our data indicate that the abnormal activation of PGRN-deficient myeloid cells might have a significant effect on the exacerbation of CNV.

We also showed that silencing PGRN in RAW264.7 cells led to an abnormal activation of the cells. In addition, hypoxic conditions promoted the production of proangiogenic and proinflammatory cytokines from PGRN-silenced macrophages and *Grn*^*+/+*^ mice-derived peritoneal macrophages (Figs. 4 and 5, Supplemental Figures 4). In addition to VEGF-A, various inflammatory factors including IL-1β, TNF-α, complement components, and CCL2 have been shown to promote pathological angiogenesis directly and indirectly [40–43]. Our results showed that the levels of expression of all these proinflammatory factors were significantly higher in siGrn- and hypoxia-exposed macrophages (Fig. 5). Of these factors, it is interesting that only CCL2 expression is significantly increased in PGRN-silenced macrophages under normoxic conditions. The interaction between CCL2 and CCR2 is one of the most important signaling for the recruitment of mononuclear phagocytes and monocyte-derived cells themselves also secrete CCL2 [44, 45]. Thus, the increase in CCL2 in PGRN-deficient macrophages might play a central role in the recruitment of Iba-1^+^ myeloid cells into the CNV region. Although anti-VEGF agents are commercially available to treat exudative AMD, several clinical trials have examined new therapeutic agents that target components of other signaling pathways. Therefore, regulation of the infiltration of Iba-1^+^ myeloid cells into the CNV area is important for the suppression of CNV formation, and this might be a new therapeutic method that can complement the shortcomings of anti-VEGF therapy.

In the PGRN-deficient macrophages, the expression level of lysosome-associated proteins and the number of activated lysosomes were significantly higher (Fig. 6, Supplemental Figures 5). Although we did not demonstrate the mechanisms by which lysosomal activation is caused by PGRN dysfunction in this study, lysosomal abnormalities in the microglia of the brain of *Grn*^*−/−*^ mice have been reported in recent studies. PGRN is localized to late endosomes and early lysosomes in wild-type microglia, and the microglia in *Grn*^*−/−*^ mice show a marked increase in the size and number of lysosomes [46]. Moreover, an earlier study reported that PGRN insufficiency induced lysosomal biogenesis in microglia and neurons [47, 48]. Abnormal activation of lysosomal proteases, such as cathepsin D and cathepsin B, results in phenotypic changes of myeloid cells through the activation of NF-κB. This activation has been shown to induce the expression of various proinflammatory genes including those for cytokines and chemokines, and it also participates in inflammasome regulation [49]. Therefore, normalization of lysosomal function in myeloid cells might be a new therapeutic target for CNV pathologies.

**Fig. 7:**
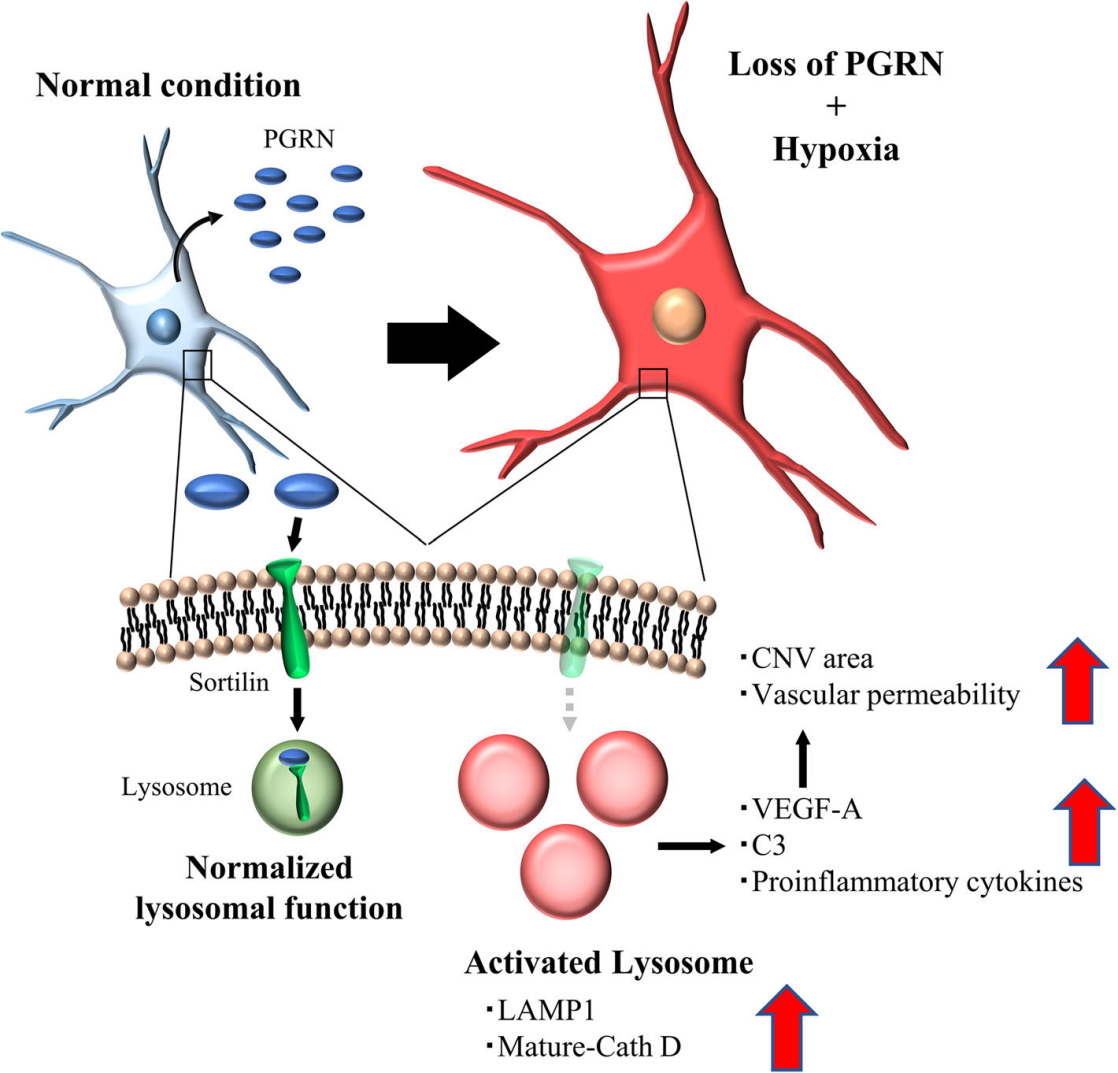
Graphical abstract. In wild-type mice, the Iba-1^+^ myeloid cells including the microglia and macrophages express high levels of PGRN in response to acute inflammation. PGRN regulates the lysosomal function and activation in Iba-1^+^ myeloid cells. However, in PGRN deficient mice, the reduced levels of PGRN lead to abnormal activation of the lysosomes in the Iba-1^+^ myeloid cells. Lysosome-activated myeloid cells express proangiogenic and proinflammatory factors and promote the formation of CNV and vascular permeability

Next, we focused on sortilin, a transmembrane receptor, that acts as a transporter of extracellular PGRN to lysosomes rather than serving as a signaling site [50]. Interestingly, the level of expression of sortilin was significantly lower in siGrn- and hypoxia-exposed macrophages (Fig. 7). The reduction of sortilin might prevent the normal transport of PGRN to lysosomes which would accelerate the abnormal activation of myeloid cells.

## Conclusion

PGRN-deficient myeloid cells have altered lysosomal function and abnormal inflammation under hypoxic stress. This leads to an exacerbation of CNV lesions in the laser-induced CNV model (Fig. 7). Increased inflammatory properties of myeloid cells associated with abnormal activation of lysosomes might play important roles in the development of CNV in patients and the regulation of these cells might be a novel therapeutic target for exudative AMD.

## Supplementary Information

**Additional file 1: Supplemental Figure 1.** The expression of PGRN in retina and RPE-choroid complex after laser photocoagulation. A. and B. PGRN expression levels in the retina and RPE-choroid-sclerafrom C57BL/6J mice at 0, 1, 3, 5, and 7 days after photocoagulation. While the expression level of PGRN in laser irradiated retina did not change significantly, a significant increase of PGRN was confirmed in the RPE-choroid-sclera complex at 3 and 5 days after the laser photocoagulation. Data are the means ± SEMs, (*n* = 5).^*^P<0.05, ^**^P<0.01 vs. Control (A: one-way ANOVA followed by Dunnett’s test, B: Dunn-Bonferroni post hoc method following a significant Kruskal-Wallis tests).

**Additional file 2: Supplemental Figure 2.** PGRN expression in CNV lesion in WT and KO mice. A. Immunofluorescent staining of CNV lesion from *Grn*^*+/+*^ and *Grn*^*−/−*^ mice with anti-PGRN (magenta) antibodies. PGRN was not expressed at the laser-irradiated sites in *Grn*^*−/−*^ mice. Nuclei and CNV are stained with Hoechst 33342 (blue) and FITC-dextran (green). Scale bars, 50 μm.

**Additional file 3: Supplemental Figure 3.** The expression and location of Iba-1 and CD68 in myeloid cells. A. Immunocytochemistry on *Grn*^+/+^ derived peritoneal macrophages with anti-Iba-1 (green) and anti-CD68 (red) antibodies. Nuclei were stained with Hoechst 33342 (blue). Scale bars, 50 μm. B. Immunohistochemistry of a laser-injured eye of C57BL/6J mice at 5 days after the photocoagulation with anti-Iba-1 (green) and anti-CD68 (red) antibodies. Nuclei were stained with Hoechst 33342 (blue). Scale bars, 50 μm.

**Additional file 4: Supplemental Figure 4.** PGRN knockdown efficiency of siRNA transfection in RAW264.7 cells. A. and B. PGRN expression level in RAW264.7 cells after transfection with siControl or three types of siGrns (*n* = 4). Data are the means ± SEMs (*n* = 4). ^**^P<0.01 vs. siControl (one-way ANOVA followed by Tukey’s tests).

**Additional file 5: Supplemental Figure 5.** Lysosomal abnormality in *Grn*^*−/−*^ mice derived peritoneal macrophages. A. Cell viability of *Grn*^*+/+*^ and *Grn*^*−/−*^ mice derived peritoneal macrophages. Data are the means ± SEMs (*n* = 6). ^**^P<0.01 vs. *Grn*^*+/+*^ (Welch’s *t*-test). B. Representative images of LysoTracker Red DND-99 staining (red) and phase contrast images of peritoneal macrophages. Scale bar: 10 μm. C. Quantitative analysis of the fluorescence intensity of LysoTracker^+^ cells. Data are the means ± SEM (*n* = 6). ^*^^*^P<0.01vs. *Grn*^*+/+*^ (Welch’s *t*-test). D. Representative images of western blots showing immunoreactivity against LAMP1, cathepsin D, and β-actin. E. F. and G. Quantitative analysis of expression level of LAMP1 (E), pre-cathepsin D (F), and mature-cathepsin D (G) in RAW264.7 cells after transfection of siControl or siGrn and exposure to hypoxia (1% O_2_, 12 h). Data are the means ± SEMs (n = 4). ^**^P<0.01 vs. *Grn*^*+/+*^ (E and F: one-way ANOVA followed by Tukey’s tests, G: Dunn-Bonferroni post hoc method following a significant Kruskal-Wallis tests).

## Data Availability

Data supporting the conclusions of this article are presented in this manuscript.

## Abbreviations

AMD: Age-related macular degeneration;
C3: Complement component 3
CCL2: C-C motif chemokine ligand 2
CCR2: C-C motif chemokine receptor 2
CD68: Cluster of differentiation 68
CNV: Choroidal neovascularization
CX3CR1: C-X3-C motif chemokine receptor 1
DMEM: Dulbecco’s modified eagle medium
FFA: Fundus fluorescein angiography
FITC: Fluorescein isothiocyanate
FTD: Frontotemporal dementia
Grn: Granulin
Iba-1: Ionized calcium binding adapter molecule 1
ICC: Immunocytochemistry
IHC: Immunohistochemistry
IL-1β: Interleukin-1 beta
iNOS: Inducible nitric oxide synthase
LAMP1: Lysosomal-associated membrane protein 1
NCL: Neuronal ceroid lipofuscinosis
PGRN: Progranulin
RPE: Retinal pigment epithelium; siRNA: Small interfering ribonucleic acid; TNF-α: Tumor necrosis factor alpha; VEGF: Vascular endothelial growth factor; VPS10: Vacuolar protein sorting 10
